# HHLA2, a member of the B7 family, is expressed in human osteosarcoma and is associated with metastases and worse survival

**DOI:** 10.1038/srep31154

**Published:** 2016-08-17

**Authors:** Pratistha Koirala, Michael E. Roth, Jonathan Gill, Jordan M. Chinai, Michelle R. Ewart, Sajida Piperdi, David S. Geller, Bang H. Hoang, Yekaterina V. Fatakhova, Maya Ghorpade, Xingxing Zang, Richard Gorlick

**Affiliations:** 1 Department of Molecular Pharmacology, Albert Einstein College of Medicine, Bronx, NY, USA; 2 Division of Pediatric Hematology, Oncology, Marrow & Blood Cell Transplantation, Children’s Hospital at Montefiore, Albert Einstein College of Medicine, Bronx, NY, USA; 3 Department of Microbiology and Immunology, Albert Einstein College of Medicine, Bronx, NY, USA; 4 Department of Pathology, Montefiore Medical Center, Albert Einstein College of Medicine, Bronx, NY, USA; 5 Department of Orthopedic Surgery, Montefiore Medical Center, Albert Einstein College of Medicine, Bronx, NY, USA; 6 Sophie Davis School of Biomedical Education, Manhattan NY, USA

## Abstract

Over the past four decades there have been minimal improvements in outcomes for patients with osteosarcoma. New targets and novel therapies are needed to improve outcomes for these patients. We sought to evaluate the prevalence and clinical significance of the newest immune checkpoint, HHLA2, in osteosarcoma. HHLA2 protein expression was evaluated in primary tumor specimens and metastatic disease using an osteosarcoma tumor microarray (TMA) (n = 62). The association of HHLA2 with the presence of tumor infiltrating lymphocytes (TILs) and five-year-event-free-survival were examined. HHLA2 was expressed in 68% of osteosarcoma tumors. HHLA2 was expressed in almost all metastatic disease specimens and was more prevalent than in primary specimens without known metastases (93% vs 53%, p = 0.02). TILs were present in 75% of all osteosarcoma specimens. Patients whose tumors were ≥25% or ≥50% HHLA2 positive had significantly worse five-year event-free-survival (33% vs 64%, p = 0.03 and 14% vs 59%, p = 0.02). Overall, we have shown that HHLA2 is expressed in the majority of osteosarcoma tumors and its expression is associated with metastatic disease and poorer survival. Along with previously reported findings that HHLA2 is a T cell co-inhibitor, these results suggest that HHLA2 may be a novel immunosuppressive mechanism within the osteosarcoma tumor microenvironment.

Osteosarcoma is the most common primary malignancy of bone^1^,^2^,^3^. Although the initial introduction of multimodal therapy has led to great improvements in osteosarcoma, patient survival rates have remained stagnant for the past four decades. Outcomes in patients with metastatic disease or recurrence have remained dismal, with a less than 20% 5-year survival rate^3^,^4^,^5^. There is a need to develop new therapies, especially those that target patients who are refractory to current treatments.

The non-silent somatic mutation rate of osteosarcoma, 1.2 mutations per megabase, is higher than that of genetically complex adult malignancies such as breast cancer and lymphoma^1^,^6^,^7^,^8^,^9^,^10^. Due to the relationship between genetic complexity and neoantigen production^7^,^8^,^9^,^11^,^12^, osteosarcoma is a promising target for immune checkpoint inhibitors^11^,^12^. There has been great interest in the B7/CD28 families of immune-regulatory ligands/receptors and their potential roles in cancer pathogenesis. The B7 family consists of three phylogenetic subgroups–1) B7-1, B7-2, and ICOS-L; 2) Programmed Death Ligand 1 (PD-L1) and 2 (PD-L2); and 3) B7-H3, B7x, and HERV-H LTR-associating 2 (HHLA2)^13^. These ligands are typically expressed on antigen presenting cells (APCs) and have the ability to modulate T-cell proliferation and function. Tumor cells are also capable of expressing members of the B7 family in order to evade immune detection^14^,^15^,^16^.

HHLA2 is the most recently identified member of the B7 family^13^,^17^,^18^,^19^. Unlike other members of the B7 family, HHLA2 is not expressed in mice or rats^13^,^18^. HHLA2 has been reported to have both co-inhibitory^13^,^20^ and co-stimulatory functions^19^. HHLA2 is expressed on peripheral blood monocytes and can be induced on B cells^13^,^19^. HHLA2 does not bind to any other members of the B7 family or their receptor CD28 family, rather it functions via binding to TMIDG2 and a potential second unknown receptor^13^,^19^,^20^,^21^,^22^. TMIDG2 is expressed on endothelial cells and may have a role in angiogenesis^22^,^23^.

HHLA2 is expressed in multiple cancers, including triple negative breast cancer (TNBC) and melanoma, but has limited expression on normal tissues^24^. In addition, HHLA2 expression was associated with the presence of lymph node metastasis and higher stage disease in patients with TNBC. To date, HHLA2 expression in osteosarcoma has not been examined. In this study, we assess the expression patterns of HHLA2 in osteosarcoma in the context of the immune microenvironment.

## Results

### Demographics

Fifty-four samples from the TMA had associated clinical data, of which 14 (26%) were specimens obtained at the time of initial biopsy, 28 (52%) were specimens obtained at the time of definitive surgery, and 13 (22%) were obtained from metastatic tumors (Table 1). An additional two specimens obtained at the time of initial biopsy, 17 specimens obtained at the time of definitive surgery, and five metastatic tumor specimens were available for analysis of HHLA2 and TILs, did not have either associated clinical outcome or demographic data.

The initial biopsy specimens were all obtained prior to chemotherapy exposure. With the exception of two patients, all the specimens obtained at the time of definitive surgery were obtained from patients who had received neo-adjuvant chemotherapy. Thirteen metastatic tumor specimens were obtained from eight unique patients.

### HHLA2 is expressed in the majority of osteosarcoma tumors

**Figure 1:**
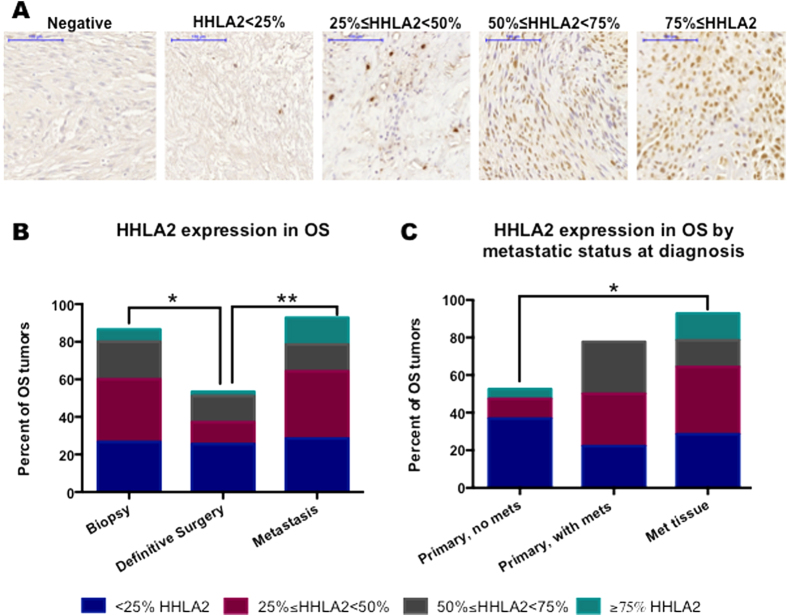
HHLA2 is expressed in the majority of osteosarcoma tumors. (**A**) representative tumors for each HHLA2 grade based on IHC. The ICH scores were based on the percentage of tumor cells that were HHHLA2 positive, with the five subdivisions being: negative, less than 25% positive, 25% ≤ HHLA2 < 50%, 51% ≤ HHLA2 < 75%, and equal or greater than 75% positive. Scale bar, 100 μM. (**B**) The majority of tumors examined were HHLA2 positive at all times of diseases progression. There is a significant decrease in HHLA2 positive cells between the time of biopsy and definitive surgery (77% vs 53%, p = 0.03). Metastatic samples have a higher percentage of cells staining positive for HHLA2 when compared to samples at definitive surgery (93% vs 53%, p = 0.01) and at biopsy (93% vs 77%, p = 0.001). (**C**) HHLA2 expression was sorted into three groups based on tissue type and presence of metastasis: primary tissue with metastasis, primary tissue without metastasis, and metastatic tissue. The percentage of cells expressing HHLA2 is significantly increased in metastatic tissue compared to primary tissue without presence of metastatic disease (53% vs 93%, p = 0.02).

HHLA2 expression was quantified as the percentage of positive tumor cells on the TMA by IHC and categorized as tumor specimens obtained at the time of initial biopsy, at the time of definitive surgery, or from metastatic disease (Fig. 1A). Overall, 68% of tumors expressed HHLA2, with 87%, 54%, and 93% expressing HHLA2 in the biopsy specimens, definitive surgery specimens, and metastatic disease specimens, respectively. Over 50% of tumors that expressed HHLA2 demonstrated an expression pattern with greater than 25% of tumor cells staining positive. The percentage of HHLA2 positive tumors was significantly lower in the definitive surgery specimens compared with the biopsy specimens (53% vs 77%, p = 0.03), as well as the metastatic specimens (53% vs 93%, p = 0.01) (Fig. 1B). HHLA2 expression was validated in a second set of osteosarcoma tumors, mostly collected at the time of definitive surgery. HHLA2 was expressed in 56% of the second set of tumors, similar to the definitive surgery samples in the TMA.

### HHLA2 expression has increased prevalence in metastatic tumor specimens

Given the high prevalence of HHLA2 expression in metastatic tissue, the relationship between HHLA2 expression in specimens from patients’ primary site of disease was compared with the presence or absence of metastatic disease. In this analysis, expression from the biopsy and definitive surgery sample sets were combined, as both cohorts represent primary osteosarcoma tissue. HHLA2 was greater in primary tumors from patients with the presence of metastatic disease compared with samples from patients without metastatic disease; however, this was not statistically significant (78% vs 53%, p = 0.17). HHLA2 expression was significantly greater in metastatic tissue compared to tumors from the primary tumor in patients without metastatic disease (93% vs 53%, p = 0.02) (Fig. 1C).

### HHLA2 and PD-L1 are co-expressed in osteosarcoma

HHLA2 expression was examined in a second cohort of patient samples, in which we previously assessed PD-L1 expression^25^. Overall, HHLA2 expression was more prevalent than PD-L1 expression (56% vs. 25%, p = 0.003), and within tumor specimens a higher proportion of cells, greater than 25% of the tumor volume, stained positive for HHLA2 compared with PD-L1 (21.4% vs 0%, p < 0.001). The vast majority of PD-L1 positive tumors were also HHLA2 positive (92% vs 8%, p = 0.006) (Figure S1). Double immunofluorescence staining demonstrated that PD-L1 was often co-expressed with HHLA2. In addition, double immunofluorescence staining confirmed that HHLA2 was expressed in a higher proportion of cells compared with PD-L1 (Figure S2).

### Tumor-infiltrating lymphocytes are prevalent in the osteosarcoma tumor microenvironment

**Figure 2:**
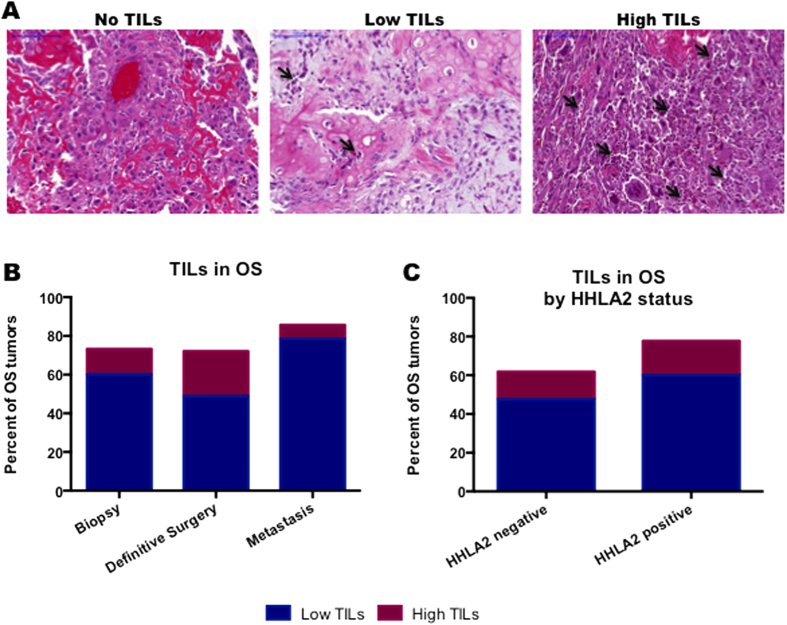
TILs are present in the majority of osteosarcoma tumors. (**A**) H&E staining was used to assess the presence of TILs in osteosarcoma samples. Samples were graded as having no TILs, low TILs (<30% TILs), or high TILs (>30% TILs). Scale bar, 100 μM. (**B**) TILs are present in the majority of osteosarcoma samples at all times of specimen collection. (**C**) TILs are present at high levels in osteosarcoma regardless of the HHLA2 status.

The presence of TILs was assessed on a hematoxylin and eosin (H&E) stained TMA (Fig. 2A). Overall, 75% of tumors demonstrated presence of TILs in the tumor microenvironment—in 73%, 72%, and 86% of biopsy samples, definitive surgery samples, and metastatic samples, respectively. The level of TILs was not associated with the tissue type (Fig. 2B).

**Figure 3:**
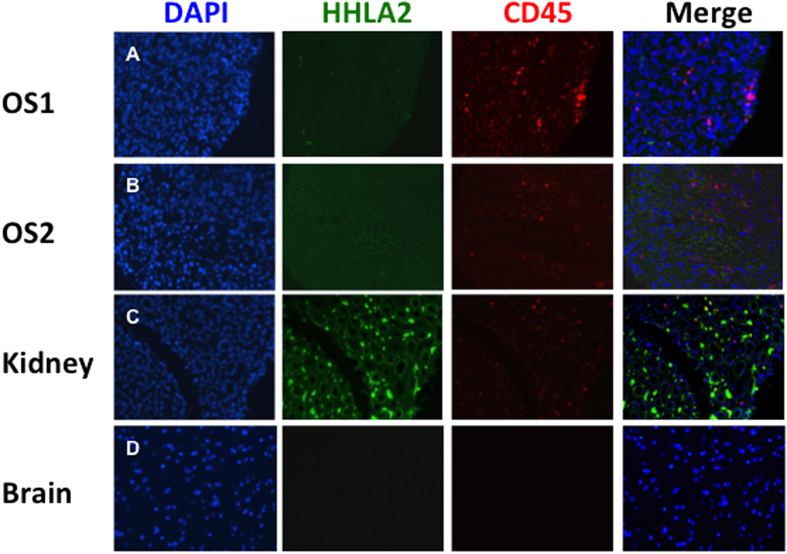
The majority of HHLA2 expression is on the tumor. (**A**) OS1 tumor demonstrated HHLA2 expression in a small area of the tumor. CD45 positive myeloid cells, which were clearly HHLA2 negative, were present. (**B**) OS2 tumor demonstrated HHLA2 expression homogenously throughout the tumor. CD45 positive myeloid cells, some of which were HHLA2 positive, were present. (**C**) Kidney tissue demonstrated HHLA2 expression in the renal tubules. Of the CD45 positive myeloid cells present, a small fraction were also HHLA2 positive. (**D**) Brain tissue was negative for both HHLA2 expression and CD45 myeloid cell infiltration.

Due to the high levels of TIL infiltration in many osteosarcoma tumors, we assessed if HHLA2 was expressed on tumor cells or on immune cells by performing double IF of HHLA2 and CD45, a myeloid cell marker. HHLA2 and CD45 were rarely co-expressed in tumor specimens. The majority of HHLA2 expression was independent of CD45, suggesting HHLA2 is most frequently expressed on non-myeloid cells, most likely on osteosarcoma cells (Fig. 3).

### HHLA2 expression is associated with tumor infiltration by specific immune cell subtypes

The relationship between HHLA2 expression and lymphocyte infiltration was assessed. The majority of both HHLA2 negative and positive tumors demonstrated the presence of TILs, 62% and 78% (p = 0.24), respectively. Both HHLA2 negative and positive tumors expressed varied levels of TILs and there was no significant association between the total level of TILs present in the microenvironment and the expression of HHLA2 (Fig. 2C). In order to clarify the association between HHLA2 and immune cell infiltration, we compared HHLA2 expression to immune infiltration in a second slide set, as determined in a previous study^25^. In this cohort, HHLA2 expression was significantly associated with the presence CD3+ T cells (70% vs 30%, p = 0.006), CD20+ B cells (78% vs 22%, p = 0.001), CD1a+ dendritic cells (78% vs 22%, p = 0.001), and CD68+ macrophages (67% vs 31%, p = 0.03) (Table 2).

### HHLA2 expression is associated with differential event free survival

**Figure 4:**
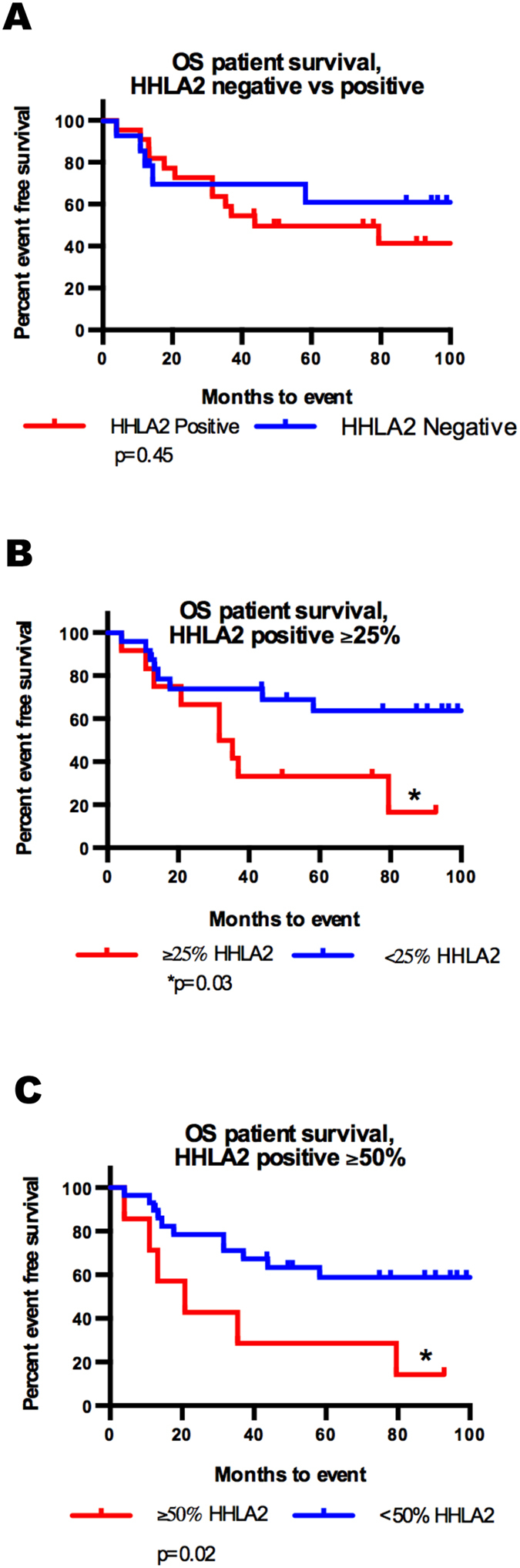
Higher percentages of HHLA2 expressing tumor cells are associated with worse survival in osteosarcoma. Kaplan-Meyer curves were generated using multiple HHLA2 positive thresholds–specifically all HHLA2 positive, greater than 26% HHLA2 positive, and greater than 51% HHLA2 positive. (**A**) HHLA2 does not appear to be significantly associated with 5-year event free survival (5-yr EPS) when the positive threshold is set to ‘greater than zero’ (50% positive vs 61% negative, p = 0.45). (**B,C**) When the thresholds for positivity are set higher there are significantly worse 5-yr EPS for patients whose tumors are greater than 25% (64% vs 33%, p = 0.03) or 51% HHLA2 positive (59% vs 14%, p = 0.02) vs those that are not.

In order to assess the prognostic relevance of HHLA2 expression in biopsy and definitive surgery, event free survival was calculated for patients based on the percentage of HHLA2 expression. Kaplan Meier curves were generated utilizing HHLA2 expression thresholds of greater than 0%, 25%, and or 50% positive. Due to the limited number of specimens that demonstrated >75% expression, Kaplan Meier curves were not generated using the >75% threshold. All survival curves demonstrated that EFS was higher in the cohort with lower or no expression of HHLA2 and achieved statistical significance utilizing the 25% and 50% thresholds (Fig. 4A–C).

Overall, previously validated risk factors in osteosarcoma were associated with worse survival in our cohort. HHLA2 expression, however, was not associated with higher age (50% vs 63%, p = 0.66), worse anatomic site (58% vs 67%, p = 0.18), metastatic status at diagnosis (67% vs 39%, p = 0.18), or histologic response to neoadjuvant chemotherapy (67% vs 36%, p = 0.54) (Figure S3). However, as previously noted HHLA2 expression was higher in metastatic tissue compared to primary tumor without metastatic disease (93% vs 53%, p = 0.02). Further analysis is needed to assess the relationship between HHLA2 expression and risk factors associated with poor prognosis in osteosarcoma

## Discussion

Here we present the first study on the expression and clinical significance of HHLA2, a member of the B7 family of immune checkpoint molecules, in osteosarcoma. We found that HHLA2 is expressed in the majority of osteosarcoma samples. A previous study examined the expression of HHLA2 in a variety of cancers, not including osteosarcoma, and demonstrated variable expression in different histologic subgroups ranging from zero to 70%^24^. The number of samples in each subgroup was small, limiting the ability to extrapolate the actual prevalence of HHLA2 expression^24^. Our results demonstrate that HHLA2 expression in osteosarcoma is on the higher end of the spectrum, similar to the frequency seen in breast cancer.

We further demonstrated that HHLA2 was more often expressed in patients with advanced disease and in metastatic tissue. It is interesting that HHLA2 expression is higher in both primary tumors with metastatic disease and in metastatic tissues compared to primary tissue without metastatic disease. Given that metastasis is an inefficient process consisting of a series of rate-limiting steps^26^, our results suggest that primary tumor cells with higher HHLA2 expression may have an increased capability to survive after leaving the primary tumor and entering the circulation. It is also possible that tumor cells may up-regulate HHLA2 expression as they form metastatic colonies in new tissues.

Although both HHLA2 and PD-L1, another B7 family member, are able to inhibit human CD4 and CD8 positive T cells functions, their expression patterns on human immune cells are very different^13^. Our results, demonstrating the high frequency of HHLA2 protein expression, mirror the prevalence of PD-L1 mRNA expression in osteosarcoma. A previous study demonstrated that, although PD-L1 mRNA is expressed in 32 of 38 patient samples, the level of gene expression did not correlate with clinical outcome^27^. However, our results revealed that an increased percentage of HHLA2 positive tumor cells was not only associated with high-risk features, but also with worse event-free survival. Taken together, these observations suggest that the HHLA2 pathway may represent a more important immunosuppressive mechanism in the human osteosarcoma tumor microenvironment.

Due to it’s genetic complexity, it is important to examine the role of multiple mechanisms of immune evasion in osteosarcoma. The highly immunogenic nature of bone may contribute to the prevalence of TILs in osteosarcoma^28^. Furthermore, the expression of the repertoire of B7 family ligands suggests that immune evasion may be an essential component of osteosarcoma oncogenesis. Consequentially, limiting treatment to targeting one component of the pathway may not be sufficient to inhibit immune escape in osteosarcoma.

Previous studies have found an association between the presence of TILs and PD-L1 expression^25^,^29^,^30^. In this study we found that TILs are present in the majority of osteosarcoma tumors, which corroborates these prior findings. However, in the case of HHLA2, there is no significant correlation between its expression and the presence of TILs, but this may be secondary to the limited numbers of HHLA2 or TIL negative tumors. In a second cohort we found that HHLA2 expression may be associated with a variety of infiltrating immune cells, including both T and B cells. It has previously been shown that HHLA2 expression can be activated in an immune dependent inflammatory fashion or via copy number amplification in an immune independent manner^22^. In the case of osteosarcoma, which is a genetically complex disease, copy number variation may be a driver of expression. Additionally, as HHLA2 has a T cell co-inhibitory role, the TILs that are present in osteosarcoma may be nonfunctional^13^. The major limitation in this study is the relatively small sample size of the cohorts analyzed. Validation of these findings will require analysis of HHLA2 expression in additional large patient cohorts.

In summary, our findings point to an additional pathway for therapeutic targeting in osteosarcoma, either directly or by inhibiting the tumors interaction with immune cells. At this time there are no inhibitors of HHLA2 available for clinical use, but preventing immune tolerance, by targeting PD-L1 or CTLA-4, in the tumor microenvironment has proven effective in a variety of other cancers. Due to the prevalence of HHLA2 expression in osteosarcoma, this pathway needs to be further explored in patients who do not respond to conventional therapies. Furthermore, targeting HHLA2 in addition to other immune checkpoint inhibitors may be required to completely inhibit the pathway and sensitize osteosarcoma to immune mediated clearance.

## Materials and Methods

### Tissue collection and tumor microarray (TMA) construction

Tumors collected from osteosarcoma patients were used to construct a TMA, as previously described^31^,^32^. The TMA contains cores from primary osteosarcoma tumors collected at the time of biopsy and definitive surgery, as well as from metastatic tissues. A pathologist reviewed the tumors in order to confirm the diagnosis of osteosarcoma. In total, the TMA contains cores from 62 samples representing 51 unique patients, of which 54 cores were usable. The cores that were determined unusable detached from the slide during processing. The TMA was constructed using 1-millimeter (mm) cores that were acquired from the formalin-fixed paraffin embedded tissue block from each individual tumor. Five-micron thick sections were cut and used for subsequent analysis^33^. A second set of osteosarcoma tumors was collected from 48 patients, mostly at the time of definitive surgery, of which 29 had associated demographic information. The set of tumors were sectioned as whole slides, as previously described^25^.

The Ethics Committees and Institutional Review Boards at Montefiore Medical Center, Memorial Sloan Kettering Cancer Center, and the Center for Cancer Research approved the study. Written informed consent was obtained from the patients and their parents/guardians prior to tissue collection. All procedures were conducted in accordance with guidelines provided by the Ethics Committees and Institutional Review Boards.

### Immunohistochemistry

IHC was optimized for proper antigen retrieval and antibody concentrations using HHLA2 transfected and CTLA-4 transfected 3T3 cells as positive and negative controls^13^,^24^, respectively. Osteosarcoma tumor slides were baked at 60 °C for one hour and antigen retrieval was performed using tri-citrate buffer, pH 8.0 at 100 °C for 20 minutes. Slides were de-paraffinized using xylene, rehydrated along an ethanol gradient, and blocked using dual endogenous enzyme-blocking reagent (Dako). Slides were then stained with a mouse anti-human HHLA2 monoclonal antibody (clone 566.1, IgG1^13^,^24^) at a 1:20 dilution for one hour at room temperature followed by one-hour room temperature incubation with a secondary biotinylated anti-mouse antibody (Abcam, ab98693). Peroxidase activity, and antigen expression in the TMA, was detected using the DakoCytomation LSAB2 system-HRP (Dako). Slides were counterstained with hematoxylin (Harris).

The whole slide samples of the second cohort were treated similarly to the TMA, with the exception of an additional normal goat serum block (Santa Cruz) prior to antibody staining. Furthermore, antigen expression in the whole slides was detected by addition of VECTASTAIN Elite ABC Kit (Vector Laboratories) and DAB (3,3-diaminobenzidine) HRP substrate (Vector Laboratories).

### Immunofluorescence (IF)

IF was optimized for proper antigen retrieval and antibody concentrations using placenta, which is positive for both HHLA2 and PD-L1. Slides were baked then underwent subsequent antigen retrieval, de-paraffinization, rehydration, and blocking in normal goat serum block (Santa Cruz) prior to antibody staining, in the same manner as for IHC. Slides were then incubated for one hour in HHLA2 primary antibody followed by a second one-hour incubation in anti-mouse Alexa Fluor 488-conjugated secondary antibody (Thermo Fisher Scientific). The slides were subsequently incubated for one hour in PD-L1 or CD45 primary antibody followed by a second one-hour incubation in anti-rabbit Alexa Fluor 594-conjugated secondary antibody (Thermo Fisher Scientific). Slides were coverslipped using VECTASHEILD Antifade Mounting Medium with DAPI (Vector).

### Quantification of IHC and TILs

Slides were scanned and graded by two independent reviewers without previous knowledge of patient outcomes or demographics. Slides were graded based on the percentage of HHLA2 positive tumor cells present in the core/slide (0 = 0% staining, 1 = < 25%, 2 = 25–50%, 3 = 50–75%, and 4 = 75–100%). If scores between the two graders were discordant by one point, the higher value was used. Rarely, when the scores were discordant by more than one, then the average was used. If present, HHLA2 staining had strong intensity in osteosarcoma tumors.

A second slide cut from the osteosarcoma TMA was stained using hematoxylin and eosin (H&E). The presence of TILs in the TMA was scored by a pathologist using a previously described three-tiered scale based on the proportion of the lymphocytes present in the tumor^34^. A score of “Low” indicated ≤30% of the core was made up of TILs and a score of “High” indicated >30%. An absence of TILs was scored to be negative.

Images of IHC and H&E staining were obtained at 20x magnification using the Pannoramic Scanner and captured using Pannoramic Viewer (3DHISTECH Ltd). Images of the IF staining were captured at 20x magnification using the EVOS^®^ RL Auto Cell Imaging System (Thermo Fisher Scientific).

### Statistical analysis

Patient demographic data from each cohort was reported as frequencies for categorical variables and means with standard deviations for continuous variables. The association between expression of HHLA2 and the timing at which the specimen was obtained, the presence of metastatic disease, and the presence of TILs was assessed using Fisher’s exact test or chi square analysis. The association between HHLA2 and PD-L1 expression was also assessed using Fisher’s exact test. Kaplan Meier curves were generated assessing the association between event free survival (EFS) and HHLA2 expression, and the Log-rank (Mantel-Cox) test was utilized (GraphPad Prism software). Survival curves were generated using expression thresholds defined as percent of the tumor that is greater than 0%, ≥25%, or ≥50% HHLA2 positive. Survival curves could not be generated using a cutoff ≥75% HHLA2 expression due to a limited number of samples in this cohort.

## Additional Information

**How to cite this article**: Koirala, P. *et al.* HHLA2, a member of the B7 family, is expressed in human osteosarcoma and is associated with metastases and worse survival. *Sci. Rep.* **6**, 31154; doi: 10.1038/srep31154 (2016).

## Supplementary Material

Supplementary Information

## Figures and Tables

**Table 1:** Patient demographic information for the tumor microarray specimens.

	Biopsy	DefinitiveSurgery	MetastaticTissue
	(n = 13)	(n = 28)	(n = 13)
Median age years (SD)	16 (22)	19 (20)	16 (4)
Huvos Response			
Good	6	15	1
Poor	6	6	7
No clinical data	1	7	5
Metastasis			
Present	7	12	13
Absent	6	15	0
No clinical data	0	1	0
Site			
Long Bone	9	15	11
Other	2	9	1
No clinical data	2	4	1

**Table 2:** HHLA2 and TIL/APC co-expression.

	HHLA2positive	HHLA2negative	p-value
*PD-L1 positive	92%	8%	p = 0.006
*CD3 infiltration	70%	30%	p = 0.0004
CD8 infiltration	63%	37%	p = 0.24
CD4 infiltration	62%	38%	p = 0.38
*CD20 infiltration	78%	22%	p = 0.001
*CD1a infiltration	78%	22%	p = 0.001
CD56 infiltration	61%	39%	p = 0.22
*CD68 infiltration	67%	31%	p = 0.03

HHLA2 expression is associated with specific immune infiltration. Tumors displaying infiltration by CD3+ T cells, CD20+ B cells, CD1a+ dendritic cells, and CD68+ macrophages were more likely to be HHLA2 positive.
