# Supplementary material for: HHLA2, a member of the B7 family, is expressed in human osteosarcoma and is associated with metastases and worse survival
Source: Sci Rep. 2016 Aug 17;6:31154. doi: 10.1038/srep31154 (PMC4987662; doi:10.1038/srep31154)
Supplement: Supplementary Information [file srep31154-s1.doc]

**HHLA2, a member of the B7 family, is expressed in human osteosarcoma and is associated with metastases and worse survival**

Pratistha Koirala^1^, Michael E. Roth^2^, Jonathan Gill^2^, Jordan M. Chinai^3^, Michelle R. Ewart^4^, Sajida Piperdi^2^, David S. Geller^2, 5^, Bang H. Hoang^5^, Yekaterina V. Fatakhova^6^, Maya Ghorpade^2^, Xingxing Zang^3^, Richard Gorlick^1, 2^

**Supplementary dataset**

**Supplemental Figure 1**


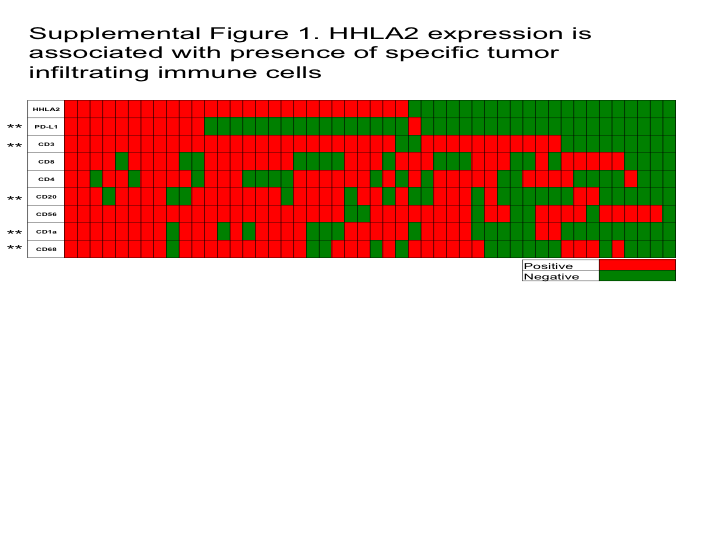


**Figure S1**. HHLA2 expression is associated with PD-L1 and presence of specific tumor infiltrating immune cells. Individual columns represent a unique patient and rows represent presence of an immune cell type. HHLA2 expression was associated with PD-L1 expression, as well as with infiltration by CD3+ T-cell, CD20+ B cells, and CD68+ macrophages. *p<0.05, **p<0.005

**Supplemental Figure 2**


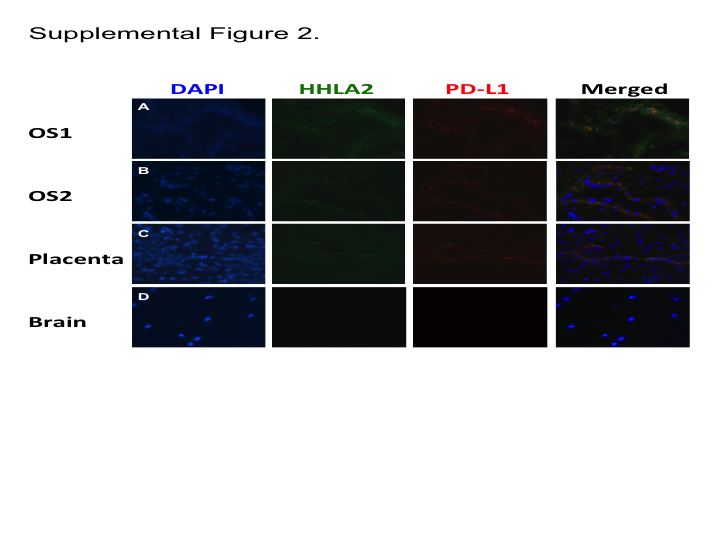


**Figure S2.** HHLA2 and PD-L1 are co-expressed in osteosarcoma. A. OS1 tumor demonstrated HHLA2 expression in the majority of the tumor. A small portion of the tumor was also PD-L1 positive. B. OS2 tumor demonstrated HHLA2 and PD-L1 co-expression in small area of the tumor. C. Placenta tissue demonstrated HHLA2 and PD-L1 co-expression. D. Brain tissue was negative for both HHLA2 and PD-L1 expression.

**Supplemental Figure 3**

**Figure S3**. HHLA2 expression is not associated with previously validated risk factors. HHLA2 expression is not significantly associated with age at diagnosis (A), anatomic site of the primary tumor (B), or metastatic status at diagnosis (C).
